# PD33 Using Environmental Impact Data To Support Health Technology Assessment At The National Institute for Health and Care Excellence: An Options Appraisal

**DOI:** 10.1017/S0266462324002964

**Published:** 2025-01-07

**Authors:** Jamie Elvidge, Juliet Kenny, Koonal Shah

## Abstract

**Introduction:**

The National Health Service (NHS) in England aims to be carbon neutral by 2045, acknowledging the link between planetary and human health. By 2028, NHS suppliers will need to report carbon footprint data for their products, including the medicines and technologies that the National Institute for Health and Care Excellence (NICE) appraises. Additionally, company level data are already being reported. This type of data may provide NICE with an opportunity to support system environmental sustainability goals.

**Methods:**

The NICE Science Policy and Research team conducted an options appraisal to consider the feasibility and acceptability of different ways NICE might engage with environmental impact data (EID). We held discussions with NICE teams that could be affected by the collection and use of EID. Discussions examined the array of potential options for NICE—from being a simple conduit for EID to incorporating EID into its decision-making—and sought to identify those that were practical for further consideration. We then discussed these options with key external stakeholders (NHS England, industry, and commissioners) to understand their expected usefulness and practicality.

**Results:**

Several options for NICE to use EID were identified as suitable for further consideration. Using company level data, NICE could encourage companies to engage with NHS sustainability goals by citing their carbon reduction plans alongside guidance or using them to prioritize its activities. Using product level data, NICE could pilot an evaluation comparing the environmental outcomes of competing health technologies that have no differential direct health outcomes. NICE could also publish environmental impact assessment tools to help commissioners consider EID in procurement decisions. Better data and methodological standards are needed before NICE might consider embedding product-level EID in its usual decision-making frameworks.

**Conclusions:**

Our options appraisal has identified several ways that NICE might start to engage with EID to have a positive impact on NHS sustainability goals and wider planetary health. Future work will scope out how these activities should proceed. The identified options are not necessarily mutually exclusive and may evolve as the data and methods around sustainable health care continue to advance.

